# Five new synonyms in *Epimedium* (Berberidaceae) from China

**DOI:** 10.3897/phytokeys.49.8768

**Published:** 2015-04-22

**Authors:** Yanjun Zhang, Haishan Dang, Shengyu Li, Jianqiang Li, Ying Wang

**Affiliations:** 1 Key Laboratory of Plant Germplasm Enhancement and Specialty Agriculture,; 2 Wuhan Botanical Garden, Chinese Academy of Sciences, Wuhan, 430074, P. R. China

**Keywords:** *Epimedium*, Berberidaceae, synonyms, China

## Abstract

Five new synonyms in Chinese *Epimedium* are designated in the present paper. *Epimedium chlorandrum* is treated as a synonym of *Epimedium acuminatum*; *Epimedium rhizomatosum* as a synonym of *Epimedium membranaceum*; *Epimedium brachyrrhizum* as a synonym of *Epimedium leptorrhizum*; *Epimedium dewuense* as a synonym of *Epimedium dolichostemon*; and Epimedium sagittatum var. oblongifoliolatum as a synonym of *Epimedium borealiguizhouense*.

## Introduction

*Epimedium* L., the largest herbaceous genus of Berberidaceae, contains approximately 58 species distributed in temperate hilly or montane regions from Japan to Algeria with enormous gaps in between. China, where *Epimedium* reaches its zenith of diversity, possesses approximately 48 species, of which are all endemic except *Epimedium koreanum* Nakai (Stearn 2002; Ying et al. 2011). The genus *Epimedium* has been insufficiently understood because of the morphological variation within and among some Chinese *Epimedium* species. As a result, some new *Epimedium* species were published which are, in fact, synonymous with existing species. In the present paper, based on extensive studies on Chinese *Epimedium* plants in herbaria, field investigations, and cultivation, we reduce the following four species and one variety: *Epimedium chlorandrum* Stearn, *Epimedium rhizomatosum* Stearn, *Epimedium brachyrrhizum* Stearn, *Epimedium dewuense* S.Z. He & W.F. Xu, and Epimedium sagittatum Maxim. var. oblongifoliolatum Z. Cheng, as new synonyms of five species of the genus.

## Materials and methods

Herbarium specimens were examined from the following herbaria: CDCM, CDBI, GZTM, HGAS, HIB, HNNU, HWA, IBK, IMD, KUN, PE, SAU, SM, and SZ. Furthermore, pertinent images of type specimens were examined from K, P, and WU. The field investigations covered all of the type localities and the typical regions of the different morphologies of the species. Additionally, plants of these species were collected and transplanted to Wuhan Botanical Garden, the Chinese Academy of Sciences, for further study.

## Taxonomic treatment

### Epimedium acuminatum

Taxon classificationPlantaeRanunculalesBerberidaceae

Franch.

Epimedium acuminatum Franch., Bull. Soc. Bot. France, 33: 109. 1886. Type: China. Guizhou, 1858, *Perny s. n.* (holotype, P!).
Epimedium chlorandrum Stearn, Kew Bull., 52(3): 660. 1997, Syn. nov. Type: China. Sichuan: Baoxing, cult. England. Hampshire: Kilmeston, Blackthorn Nursery, Apr. 1996, *Ogisu 94003* (holotype, K!).

#### Description.

Herbs 25–80 cm tall. Rhizome compact, sometimes long-creeping, 2–5 mm in diam. Leaves basal and cauline, usually trifoliolate or occasionally unifoliolate; leaflets of trifoliolate leaves narrowly ovate to lanceolate, 3–18 × 1.5–7 cm, apex long acuminate, base cordate with lobes rounded or acute, those of the lateral leaflets very unequal; unifoliolate leaves ovate or broadly ovate, 8.7–20 × 6.8–11.5 cm, apex acuminate, base cordate with lobes equal, rounded or rarely acute; leaves leathery when mature, margin spinous-serrate with the spines 1–2 mm long, adaxially deep green, glossy, abaxially glaucous, papillose, with dense or sparse short appressed stout bristle-like hairs and sometimes densely sericeous. Flowering stem usually bearing 2 opposite trifoliolate leaves, less often with 3 whorled trifoliolate leaves or 2 opposite unifoliolate leaves, rarely with 2 opposite leaves with one trifoliolate and the other unifoliolate or 3 whorled unifoliolate leaves. Inflorescence compound with lower peduncles 2–5-flowered, loose few- or many-(10–55)-flowered, glabrous or occasionally glandular hairy; pedicels 1–4 cm. Flowers 3–5 cm in diam., yellow, rose-purple, pale violet, or white. Outer sepals blunt, outer pair ovate-oblong, ca. 3 × 2 mm, inner pair broadly obovate, ca. 4.5 × 4 mm. Inner sepals ovate-elliptic, 8–12 × 3–7 mm, apex acute. Petals much longer than inner sepals, horn-shaped, tapering from the swollen but lamina-less base, curving outwards, 1.5–2.5 cm. Stamens 3–4 mm; anthers yellow or green, ca. 2.5 mm, pollen yellow or green. Capsules ca. 2 cm.

#### Distribution and habitat.

*Epimedium acuminatum* is widely distributed in Sichuan, Chongqing, Guizhou, and northern Yunnan. It usually occurs in forests, thickets, bamboo forests, and weedy slopes ranging from 270 to 2400 m in elevation.

#### Phenology.

*Epimedium acuminatum* flowers from April to May, and fruits from May to June.

#### IUCN Red List category.

Although *Epimedium acuminatum* has a relatively wide distribution in China; it should be designated as Vulnerable (VU) (IUCN 2013), because of exploitation for medicinal use, especially in Guizhou.

#### Notes.

*Epimedium acuminatum* is one of the most widespread species in the genus, and exhibits much variation in morphology; therefore, it is not surprising that there are several synonyms associated with it. Léveillé (1909) published *Epimedium komarovii* H. Lév. upon only contrasting it with *Epimedium sagittatum* Maxim., Stearn (1938) found that it was not distinct from *Epimedium acuminatum* and reduced it into synonymy. *Epimedium simplicifolium* T.S. Ying was described by Ying (1975), only separating it from *Epimedium acuminatum* in having unifoliolate, sericeous leaves. Upon extensive specimen examination, however, Zhang et al. (2011) found that the diagnostic characters of *Epimedium simplicifolium* were within the range of morphological variations of *Epimedium acuminatum* and placed *Epimedium simplicifolium* into synonymy.

*Epimedium chlorandrum* was described by Stearn (1997) as having the inner sepals being slightly ascending, and not closely appressed to the petals, and the anthers and pollen as being green. However, observations from the type locality of *Epimedium chlorandrum* show that the diagnostic character of the inner sepals as not closely appressed to the petals is not stable, and *Epimedium acuminatum* is also found having inner sepals being slightly ascending, with green anthers and pollen. There is no difference in morphology for the two species, thus, *Epimedium chlorandrum* is here treated as a new synonym of *Epimedium acuminatum*. Additionally, extensive specimen examination and field investigation demonstrate that it is incorrect for Stearn (1997) to recognize the color of anther and pollen as diagnostic features in *Epimedium* because, besides *Epimedium acuminatum*, other *Epimedium* species (e.g. *Epimedium sagittatum*, *Epimedium sutchuenense* Franch., *Epimedium elongatum* Kom., and *Epimedium leptorrhizum* Stearn) with yellow anthers and pollen are also found with green anthers and pollen.

#### Specimens examined.

**China. Chongqing:** fuling, *Fuling Med. Pl. Exped. 337* (SM), *358* (SM), *Y.L. Cao & F.D. Pu* 1291 (CDBI); Hechuan, *Hechuan Med. Pl. Exped. 193* (SM), *T.H. Tu* 5434 (PE); Jiangjin, *Jiangjin Med. Pl. Exped. 237* (SM), *Sichuan Econ. Pl. Exped. Jiang 30* (KUN, SM); Mt. Jinyun, *Z. He* et al. *1003* (SZ), *T.C. Pan & G.F. Wu 105* (PE); Nanchuan, *C.H. Hsiung 90021* (HIB, HWA, PE, SZ), *90058* (HIB, HWA, PE, SZ), *90331* (HWA, PE, SZ), *91045* (HWA, KUN, PE, SZ), *C.L. Li N1* (IMD), *N4* (IMD), *N6* (IMD), *F.T. Wang 10554* (PE), *G.F. Li 60306* (HIB, KUN, PE, SZ), *60324* (HIB, KUN, PE, SZ), *60358* (HIB, PE, SZ), *60411* (HIB, KUN, PE, SZ), *60514* (HIB, KUN, PE, SZ), *60923* (HIB, KUN, PE, SZ), *61022* (PE, SZ), *61255* (HIB, KUN, SZ), *61470* (PE, SZ), *L. Lin & B.L. Li 58* (IMD), *Mt. Jinfo Exped. 202* (PE), *N.L. Chü 1057* (PE, SZ), *S.C. Chen & K.Y. Lang 2104* (PE), *2164* (PE), *2192* (PE), *S.X. Tan* 172 (PE), *Sichuan Veg. Exped. 165* (PE, SM), *273* (CDBI, PE, SM), *T.H. Tu 2763* (PE), *3116* (PE), *W.P. Fang 802* (PE), *Z.D. Chen* et al. *960108* (PE), *Z.H. Wan & C.Y. Chu 177* (IMD), *Z. Xia 33* (CDBI), *Z.Y. Liu 15500* (PE), *15574* (PE); Wulong, *B.L. Guo 523* (IMD), *S.Z. Zhu 1562* (SM). **Guizhou:** Anlong, *Guizhou Exped. 2469* (PE, HGAS), *B.L. Guo A70* (IMD), *A73* (IMD); Anshun, *Anshun Med. Pl. Exped. 146* (GZTM); Bijie, *X. Yang s.n.* (GZTM); Chishui, *B.L. Guo A77* (IMD), *Y.M. Wang 87-273* (GZTM); Dafang, *B.L. Guo 601* (IMD), *D.L. Yu & C.L. Liu 191-5* (GZTM), *Dafang Exped. 108* (HGAS), *Y.J. Zhang 402* (HIB); Daozhen, *Anon. 85-463* (GZTM), *85-185* (GZTM), *85-2069* (GZTM), *85-60013* (GZTM), *J.M. Yuan 31* (HGAS); Dushan, *Libo Exped. 1688* (HGAS); Fuquan, *J.Y. Li 59-2* (GZTM), *59-4* (GZTM); Guanling, *Guanling Exped. 146* (GZTM); Guiding, *B.L. Guo A90* (IMD); Guiyang, *Anon. 20* (HGAS), *B. Gu s.n.* (GZTM), *C. Sun 96003* (GZTM), *G.Z. Fan 6-1* (GZTM), *6-2* (GZTM), *6-3* (GZTM), *6-4* (GZTM), *6-5* (GZTM), *L. Lin & B.L. Li 10* (IMD), *M.Z. Yang 810061* (HGAS), *810079* (HGAS), *810138* (HGAS), *P. Zhao 762* (HGAS), *807* (HGAS), *S. Guizhou Exped. 42* (HGAS, KUN, PE), *S.Y. Xiao & X.W. Li 86079* (GZTM), *S.Z. He 90001* (GZTM); Hezhang, *Hezhang Exped. 135* (GZTM); Huishui, *G.M. Qin 37* (GZTM); Jiangkou, *L.X. Yang 203* (GZTM); Kaili, *S.Z. He 90010* (GZTM), *90011* (GZTM); Kaiyang, *Kaiyang Med. Exped. 166* (GZTM), *S.Z. He 90018* (GZTM); Liuzhi, *B.L. Guo A79* (IMD), *P. Su 156* (GZTM); Longli, *M.X. wan 403031* (GZTM); Luodian, *Y.J. Zhang 426* (HIB), *427* (HIB), *428* (HIB); Nayong, *C.J. Li 86-010* (GZTM); Panxian, *Z.X. Pan s.n.* (GZTM); Qianxi, *Z.X. Wang & H.P. Xiang 7* (GZTM); Shiqian, *S.H. Xu 196* (GZTM); Shuicheng, *G.Z. Fan 9-10* (GZTM), *9-11* (GZTM); Songtao, *B.L. Guo A75* (IMD); Suiyang, *G.Z. Fan* et al. *9106* (GZTM), *9107* (GZTM), *S.F. Li s.n.* (GZTM), *S.Z. He & B. Gu 96410* (GZTM, PE), *Y.J. Chen 86-007* (GZTM); Tongzi, *B.L. Guo A13* (IMD), *X.P. Wang & P. Zhao 870212* (HGAS), *X.J. Zhang & Y.Q. Xu 97* (HIB), *Y.J. Zhang 422* (HIB), *Y. Tsiang 4994* (PE); Wuchuan, *Wuchuan Med. Exped. 870112* (GZTM), *Y.J. Zhang 164* (HIB), *166* (HIB); Xifeng, *S.Z. He 90012* (GZTM), *T.H. Yang 146* (GZTM); Xishui, *Anon. 279* (HGAS), *B.L. Guo A88* (IMD), *G.Z. Fan 9105* (GZTM), *9106* (GZTM), *9107* (GZTM), *9108* (GZTM), *9109* (GZTM), *9114* (GZTM); Xingyi, *B.L. Guo A95* (IMD), *T.X. Chen & L. Chen 106* (GZTM); Yinjiang, *X. Tang 172* (GZTM), *173* (GZTM), *Z.S. Zhang* et al. *401131* (PE, HGAS); Zhenfeng, *S.Z. He 9101* (GZTM); Zhenning, *S.Z. He & Y. Huang 9107* (GZTM), *Zhenning Exped. 156* (GZTM); Zheng’an, *J.M. Yuan 3* (HGAS), *4* (HGAS), *5* (HGAS), *6* (HGAS), *Q.H. Chen & T.L. Xu 9411* (HGAS); Ziyun, *B.L. Guo A67* (IMD), *A84* (IMD), *S.Z. He s.n.* (GZTM); Zunyi, *P.C. Tsoong 355* (KUN, PE). **Sichuan:** Baoxing, *Baoxing Med. Pl. Exped. 78-123* (SM), *Y.J. Zhang 386* (HIB), *387* (HIB), *388* (HIB), *389* (HIB); Changning, *Anon. 199* (SM); Chongzhou, *Anon. 590* (SM); Dujiangyan, *T.N. Liou 10060* (PE); Gaoxian, *Anon. 317* (SM); Gongxian, *Anon. 165* (SM); Guang’an, *Sichuan Econ. P1. Exp. Nan 127* (PE); Gulin, *C.Y. Pan & J.H. Chen 4470* (SM), *Gulin Exped. 589* (SM); Hongya, *B.L. Guo & K.W. Bao 97004* (IMD), *Hongya Exped. 952* (SM), *Sichuan Econ. Pl. Exped. Le 13* (KUN), *W.K. Bao 2298* (CDBI), *Y.J. Zhang 390* (HIB); Jiajiang, *Sichuan Econ. Pl. Exped. Le 8164* (PE, SM); Jiang’an, *Anon. 117* (SM), *K.Y. Lang 3002* (PE), *3061* (PE), *3069* (PE); Junlian, *Anon. 536* (SM); Leibo, *M.Y. He & C.M. Tan 116965* (SZ); Lushan, *G.Y. Zhong 1988-14* (SM), *1988-16* (SM), *Lushan Med. Pl. Exped. 78-398* (SM), *Y.J. Zhang 383* (HIB), *384* (HIB), *385* (HIB); Mabian, *D.Y. Hong* et al. *P.B84046* (PE), *Mabian Exped. 168* (SM); Meigu, *Sichuan Econ. Pl. Exped. Nan 6001* (PE), *Z.W. Yao 3794* (PE); Mingshan, *Mingshan Exped. 16* (SM); Mt. Omei, *B.L. Guo 88091* (IMD), *Bio. Dep., Sichuan Univ. 54207* (HIB), *C.H. Hsiung* et al. *30469* (PE), *33481* (PE), *C.H. Li 97-301* (PE), *C.S. Cheng 377* (KUN), *C.Y. Chu 331* (IMD), *D.Z. Fu 84262* (PE), *F.T. Wang 23329* (PE), *G.Y. Zhong 1988-25* (SM), *H.G. Xu 89463* (IMD), *89464* (IMD), *J.L. Hao 548* (IMD), *K.H. Yang 54343* (KUN, PE, SZ), *54207* (KUN, PE, SZ), *L.W. Wang & Z.Y. Zhang 824* (PE), *L.Z. Hu & P.Q. Duan 57-166* (SZ), *No. 236-Sichuan Exped*. *201* (PE), *Mt. Omei Exped. 176* (SM), *P. Luo* et al. *1859* (SZ), *S.L. Sun 111* (HWA, KUN, SZ), *1405* (KUN, SZ), *1566* (SZ), *1615* (SZ), *2489* (KUN, SZ), *S.S. Chien 5499* (SZ), *S.X. Wang 401* (CDBI, PE), *S.Y. Chen* et al. *3033* (SM, SZ), *3080* (SM, SZ), *S.Z. Guo 403* (PE), *Sichuan Econ. Pl. Exped. Le 210* (PE), *Sichuan Med. Pl. Exped. 12121* (IMD), *Sichuan Veg. Exped. 477* (CDBI, PE), *Sino-Russia Exped. 1996* (PE), *2169* (PE), *T.H. Tu 42* (PE, SZ), *1902* (PE), *T.T. Yu 312* (PE), *W.C. Cheng 10151* (KUN), *10174* (KUN, SZ), *W.P. Fang 2138* (SZ), *14692* (HWA, KUN, SZ), *15803* (SZ), *15985* (SZ), *16038* (SZ), *16134* (SZ), *16411* (SZ), *16242* (SZ), *16793* (SZ), *18299* (HWA, SZ), *18338* (SZ), *18400* (SZ), *18583* (SZ), *T.C. Lee 4391* (SZ), *4424* (KUN, SZ), *X.B. Peng 6108* (PE), *Y.H. Tao 53876* (SZ), *53927* (SZ), *838* (PE), *Y.J. Zhang 398* (HIB), *399* (HIB), *412* (HIB), *414* (HIB), 444(HIB), *Y.X. Xiao 48108* (SZ), *48742* (SZ), *Y.Y. Wang* et al. *604012* (GZTM), *604013* (GZTM), *604014* (GZTM); Pingshan, *B.L. Guo 617* (IMD), *Bio. Dep., Sichuan Univ. 110147* (SZ); Qionglai, *Anon. 233* (SM); Tianquan, *D.Y. Peng 47070* (CDBI), *47073* (CDBI); Xingwen, *Sichuan Econ. Pl. Exped. Yi 1256* (KUN, PE), *Xingwen Exped. 77-162* (SM); Xu’yong, *B.L. Guo 602* (IMD), *L.S. Chen s.n.* (IMD), *G.Y. Zhong 1988-29* (SM), *M.F. Zhong & S.G. Tang 138* (SM), *Xu’yong Exped. 86* (SM), *Y.J. Zhang 401* (HIB); Ya’an, *G.H. Tang 65-7* (SM), *Y.J. Zhang 308* (HIB), *Y.Y. Wang* et al. *604008* (GZTM), *Ya’an Exped. 78-34* (SM), *554* (SM); Yongjing, *B.L. Guo 615* (IMD), *Yongjing Exped. 78-8* (SM). **Yunnan:** Weixin, *P. Huo 1076* (KUN); Yiliang, *Z.Y. Wu 60* (KUN).

### Epimedium membranaceum

Taxon classificationPlantaeRanunculalesBerberidaceae

K. Mey.

Epimedium membranaceum K. Mey., Repert. Spec. Nov. Regni Veg. Beih., 12: 380. 1922. Type: China. Sichuan: Dujiangyan, *Limpricht 1293* (isotypes, K!, WU!).
Epimedium rhizomatosum Stearn, Kew Bull., 53(1): 226. 1998. Syn. nov. Type: China. Sichuan: Leibo, Selenggong, alt. 2040 m, cult. England. Hampshire: Kilmeston, Blackthorn Nursery, July 1997, *Ogisu 92114* (holotype, K!).

#### Description.

Herbs 20–65 cm tall. Rhizome compact or elongated. Leaves basal or cauline, trifoliolate; leaflets broadly ovate or narrowly ovate, 4–6 × 2–3 cm, apex acute or acuminate, margin with spines 1–1.5 mm, base cordate with lobes rounded or acute, those of lateral leaflets conspicuously oblique, subleathery, adaxially glabrous, abaxially glaucous, with scattered minute erect hairs. Flowering stem with 2 opposite or alternate leaves. Inflorescence paniculate, 9–40 cm long, 5–35-flowered, glandular; pedicels 1.5–2 cm. Flowers yellow, 4–6 cm in diam. Outer sepals green with base purplish. Inner sepals red, ovate-elliptic or narrowly ovate, 6–7 × 2.5–3 cm, apex acute. Petals pale yellow, much longer than inner sepals, subulate, 1.5–3.5 cm, lamina-less base. Stamens ca. 4 mm; anthers ca. 3 mm, yellow. Capsules ca. 2.5 cm.

#### Distribution and habitat.

*Epimedium membranaceum* occurs in montane forests and thickets of Sichuan and the adjoining region of northern Yunnan, at elevations of 1200 to 2500 m.

#### Phenology.

*Epimedium membranaceum* flowers from April to June, and fruits from May to July.

#### IUCN Red List category.

*Epimedium membranaceum* should be designated as Least concern (LC) according to IUCN Red List criteria (IUCN 2013).

#### Notes.

Ying (1975) placed *Epimedium membranaceum* into synonymy of *Epimedium davidii* Franch., which was adopted by the Flora Reipublicae Popularis Sinicae (Ying 2001) and the Flora of China (Ying et al. 2011). However, the two species can be easily distinguished by petal shape, with the petals of *Epimedium davidii* bearing obvious lamina while those of *Epimedium membranaceum* have no lamina. Additionally, *Epimedium davidii* was treated as the type species of series *Davidianae* Stearn while *Epimedium membranaceum* was referred to series *Dolichocerae* Stearn in the updated taxonomic system of *Epimedium* (Stearn 2002).

*Epimedium rhizomatosum* is much like *Epimedium membranaceum*. In the protologue for *Epimedium rhizomatosum*, Stearn (1998) distinguished it from *Epimedium membranaceum* in rhizome shape, closeness of spines along the leaflet margin, inflorescence morphology, and number of flowers. *Epimedium membranaceum* had a compact rhizome with annual-growth shoots 1–2 cm long while *Epimedium rhizomatosum* had an elongated rhizome and annual-growth shoots about 3–5 cm long; the leaflets of *Epimedium membranaceum* had about 9–11 spines to 3 cm of leaflet margin whereas leaflets of the same size in *Epimedium rhizomatosum* had about 15–17 spines to 3 cm of margin; *Epimedium membranaceum* had a 30–40 cm long inflorescence with numerous well-separated flowers whereas *Epimedium rhizomatosum* had a much shorter inflorescence with fewer, more crowded flowers. However, based on extensive investigation in herbaria and the field, it was found that *Epimedium membranaceum* continuously varies in the morphology of these organs and the diagnostic features of *Epimedium rhizomatosum* can fall into the variation range of *Epimedium membranaceum*. For instance, *Epimedium membranaceum* not only has compact but also slender and elongated rhizome in the type locality (even in one collection *X.J. He* et al. *131825* (SZ)).Therefore, *Epimedium rhizomatosum* is here reduced as a new synonym of *Epimedium membranaceum*.

#### Specimens examined.

**China. Sichuan:** Beichuan, *Sichuan Econ. Pl. Exped. Mian 530* (SAU, SZ), *1149* (KUN, SM, SZ), *S. Jiang* et al. 7050 (PE); Butuo, *Butuo Med. Pl. Exped. 19* (SM); Dechang, *Grade 1974, SW Normal Univ. 12067* (CDBI, HWA, PE); Dujiangyan, *B.L. Guo 603* (IMD), *604* (IMD), *Z. He 12229* (HWA, PE), *F.T. Wang 20851* (KUN, PE), *X.J. He* et al. *131825* (SZ), *Y.J. Zhang 391* (HIB); Huidong, *Huidong Med. Pl. Exped. 1* (SM), *S.K. Wu 1433* (KUN, SM, SAU, SZ); Jiangyou, *Jiangyou Med. Pl. Exped. 223* (SM); Jinyang, *B.L. Guo 612* (IMD), *Jinyang Med. Pl. Exped. 440* (SM), *Y.J. Zhang 307* (HIB); Leibo, *M.Y. Fang 11740* (SZ), *S. Jiang* et al. *7534* (KUN, PE, SZ), *Sichuan Econ. Pl. Exped. Liang 26* (PE, KUN), *Sichuan Med. Pl. Exped. 27948* (SM), *K.P. Yin 83* (SZ), *Y.J. Zhang 303* (HIB); Maoxian, *B.L. Guo 529* (IMD), *88187* (IMD), *B.L. Guo & W.K. Bao 97017* (IMD), *C. Zhang* et al. *2004010* (PE), *Maowen Med. Pl. Exped. 183* (SM), *275* (SM), *S.H. Ma 65-331* (CDCM), *Sichuan Econ. Pl. Exped. E 166* (IMD), *5189* (CDBI, KUN, SAU, SM), *Z.L. Shen 89024* (IMD); Meigu, *Meigu Med. Pl. Exped. 154* (SM), *Sichuan Econ. Pl. Exped. Liang 6001* (PE); Ningnan, *Ningnan Med. Pl. Exped. 254* (SM); Pengzhou, *G.H. Jiang 200606001* (SM), *Q. Wang* et al. *yc013* (SZ), *yc017* (SZ), *W.Z. Zeng 25* (CDCM), *X.M. Lu 18* (CDCM); Pingwu, *Sichuan Econ. Pl. Exped. Mian 2195* (SM), *X.L. Jiang 10231* (PE, SZ); Qingchuan, *Qingchuan Exped. 594* (SM), *595* (SM), *Qingchuan Med. Pl. Exped. 2222* (SM), *3130* (SM); Shifang, *B.L. Guo 536* (IMD), *X.J. He* et al. *138339* (SZ), *Shifang Exped. 118* (SM); Wanyuan, *Sichuan Econ. Pl. Exped. Da 2323* (SAU); Xichang, *Q.E. Yang 93032* (PE); Yanbian, *Yanbian Exped. 295* (SM); Yanyuan, *Yanyuan Exped. 218* (SM); Zhaojue, *Zhaojue Exped. 90* (SM). **Yunnan:** Dongchuan, *S.B. Lan 250* (PE); Kunming, *J.H. Chen 679* (SM); Lijiang, *Y.Z. Zhao 20510* (KUN); Suijiang, *B.X. Sun* et al. *233* (PE, KUN); Weixi, *K.M. Feng 3510* (PE, KUN), *3383* (PE, KUN), *4371* (PE, KUN), *Qing-Zhi Exped. 6300* (PE, KUN), *6595* (PE, KUN), *Q.W. Wang 63581* (PE, KUN), *63697* (PE, KUN), *64285* (PE).

### Epimedium leptorrhizum

Taxon classificationPlantaeRanunculalesBerberidaceae

Stearn

Epimedium leptorrhizum Stearn, J. Bot., 71: 343. 1933. Type: China. Guizhou: Guiyang, *Bodinier 2184* (holotype, P!).
Epimedium brachyrrhizum Stearn, Kew Bull., 52(3): 659. 1997. Syn. nov. Type: China. Guizhou: Fanjingshan Mts., cult. USA. Massachusetts: Hubbardston, *Darrell Probst CPC 94.0495* (holotype, K!).

#### Description.

Herbs 12–30 cm tall. Rhizome long-creeping, occasionally clump-forming, 1–2 mm in diam. Leaves basal and cauline, trifoliolate or occasionally unifoliolate; leaflets of trifoliolate leaves narrowly ovate or ovate, 3–10 × 2–5 cm, apex long acuminate, base deeply cordate with usually rounded lobes nearly touching, those of the lateral leaflets very unequal; unifoliolate leaves ovate or broadly ovate, 8–13.7 × 5–11 cm, apex acuminate, base cordate with lobes equal, rounded and rarely acute; leaves leathery, margin spinous-serrate, adaxially deep green, glossy, abaxially glaucous, papillose, and reddish pubescent along veins, especially dense at insertion of petioles and petiolules. Flowering stem with 1 leaf or 2 opposite leaves. Inflorescence racemose, 12–25 cm long, 4–12-flowered, glandular; pedicels 1–2.5 cm. Flowers ca. 4 cm in diam., white, tinged with rose or deep rose. Outer sepals green or purplish, outer pair ovate-oblong, 3–4 × ca. 2 mm, apex obtuse, inner pair broadly ovate, 4–5.5 × 3–4.5 mm, apex obtuse. Inner sepals white or pale rose, narrowly elliptic or lanceolate, 11–22 × 4–7 mm, apex acuminate. Petals slightly longer than inner sepals, almost white with base rose or deep rose, horn-shaped, up to 2.6 cm, tapering from the swollen but lamina-less base. Stamens ca. 4 mm, anthers ca. 3 mm, yellow or green. Capsules oblong, 1.5–2 cm.

#### Distribution and habitat.

*Epimedium leptorrhizum* occurs in montane forests or thickets in Chongqing, Guangxi, Guizhou, Hubei and Hunan, at elevations of 600 to 1500 m.

#### Phenology.

*Epimedium leptorrhizum* flowers from April to May, and fruits from May to June.

#### IUCN Red List category.

*Epimedium leptorrhizum* should be designated as nearly threatened (NT) according to IUCN Red List criteria (IUCN 2013), because of exploitation for medicinal use.

#### Notes.

Stearn (1997) published *Epimedium brachyrrhizum*, which had been only known from the type locality. In the protologue for *Epimedium brachyrrhizum*, the major difference between *Epimedium leptorrhizum* and *Epimedium brachyrrhizum* was that the former had a very slender elongated rhizome while the latter bore a more compact clump-forming rhizome. However, examination of a series of *Epimedium leptorrhizum* specimens shows that its rhizome is often slender and long-creeping but occasionally thicker and compact. In addition, we observed *Epimedium leptorrhizum* and *Epimedium brachyrrhizum* in herbaria, the field, and gardens, and did not observe any associated floral or foliar differences between the two species. *Epimedium brachyrrhizum* is reduced here as a new synonym of *Epimedium leptorrhizum*.

#### Specimens examined.

**China. Chongqing:** Fengdu, *Anon. 109* (SM), *247* (SM); Shizhu, *Anon. 135* (SM), *202* (SM), *Y.J. Zhang 30* (HIB), *31* (HIB), *32* (HIB); Youyang, *Anon. 216* (SM), *503* (SM), *1510* (SM); Zhongxian, *J.X. Shi 637* (HWA). **Guangxi:** Quanxian, *Y.C. Chen 102* (IBK). **Guizhou:** Anshun, *Anshun Exped. 1548* (HGAS); Guiyang, *Anon. 45* (HGAS), *635* (IMD), *B.L. Guo 94018* (IMD), *S. Guizhou Exped. 77* (HGAS, PE, KUN); Jiangkou, *S.Z. He 90005* (HGAS); Kaili, *Y.J. Zhang 156* (HIB), *435* (HIB); Longli, *B.L. Guo A38* (IMD), *A81* (IMD), *D.Q. Zhang 19* (GZTM), *Y.Y. Wang 403030* (GZTM); Luodian, *Y.J. Zhang 429* (HIB); Meitan, *Anon. 254* (IMD), *Guizhou Med. Pl. Exped. 37* (IMD); Pingba, *Anshun Exped. 1548* (PE); Songtao, *B.L. Guo A87* (IMD), *A98* (IMD), *Y.J. Zhang 174* (HIB), *177* (HIB), *298* (HIB); Suiyang, *P. Zhao 452* (HGAS); Tongzi, *Y.K. Li 11171* (HAGS); Wuchuan, *J.M. Yuan & S.L. Yang 1* (HGAS), *2* (HGAS), *Y.J. Zhang 168* (HIB), *172* (HIB); Xifeng, *B.L. Guo A104* (IMD); Yinjiang, *Anon. 680* (HGAS), *S.Z. He s.n.* (IMD), *Y.J. Zhang 159* (HIB), *Z.S. Zhang* et al. *401219* (HGAS, PE). **Hubei:** *Enshi, H.J. Li 8738* (PE, HIB), *Y.J. Zhang 13* (HIB), *14* (HIB), *15* (HIB), *16* (HIB), *18* (HIB), *71* (HIB), *141* (HIB), *142* (HIB), *143* (HIB), *144* (HIB), *145* (HIB), *146* (HIB), *147* (HIB); Hefeng, *Y.J. Zhang 64* (HIB); Laifeng, *B.L. Guo A05* (IMD); Lichuan, *B.L. Guo & X.Z. Luo 89005* (IMD), *89009* (IMD), *A47* (IMD), *A52* (IMD), *A54* (IMD), *H.J. Li 11020* (HIB), *Y.J. Zhang 22* (HIB), *23* (HIB), *24* (HIB), *25* (HIB), *26* (HIB), *27* (HIB), *29* (HIB), *30* (HIB), *64* (HIB), *73* (HIB), *Z.C. Ye 25* (HIB), *Z.E. Zhao* 32*26* (HIB), *3237* (HIB), *3238* (HIB), *3240* (HIB), *9053* (HIB); Xianfeng, *Y.M. Wang 6657* (HIB), *X.S. Zou 74010* (HIB); Xuan’en, *Y.J. Zhang 416* (HIB). **Hunan:** Baojing, *B.M. Yang 34B* (HNNU), *Y.J. Zhang 180* (HIB); Longshan, *B.L. Guo A99* (IMD); Sangzhi, *B.L. Guo A32* (IMD), *Sangzhi Insititute of Forestry Scinece 202* (KUN), *263* (KUN), *Hunan-Guizhou Exped. 3469* (KUN), *Y.J. Zhang 189* (HIB).

### Epimedium dolichostemon

Taxon classificationPlantaeRanunculalesBerberidaceae

Stearn

Epimedium dolichostemon Stearn, Kew Bull., 45(4): 685. 1990. Type: China. Chongqing: Shizhu, alt. 150 m, *Ogisu s. n.* (holotype, K!).
Epimedium dewuense S.Z. He, Probst & W.F. Xu, Acta Bot. Yunnan. 25(3): 281. 2003. Syn. nov. Type: China. Guizhou: Dejiang, in thickets on slopes, alt. 1350 m, 19 Apr. 2002, *S.Z. He* & *W.F. Xu 2419* (holotype, GZTM!; isotype, KUN!).

#### Description.

Herbs 30–50 cm tall. Rhizome compact. Leaves basal or cauline, trifoliolate; leaflets narrowly ovate or lanceolate, 8–10 × 3–4.5 cm, apex long acuminate, margin spinous-serrate with the spines 0.5–1.5 mm, base deeply cordate with lobes acute or rounded, those of lateral leaflets very oblique, leathery, adaxially glabrous or pubescent, abaxially glaucous, glabrous or pubescent. Flowering stem with 2 opposite or rarely alternate leaves. Inflorescence paniculate, 15–20 cm long, 35–70-flowered, glabrous or glandular pubescent; pedicels 1–3 cm. Flowers ca. 2 cm in diam. with spreading inner sepals. Outer sepals purplish with margin white, outer pair ovate-oblong, 3.5–4.5 × 1.2–2.5 mm, apex obtuse, inner pair ovate, 4–5.5 × 2.8–3.5 mm, apex obtuse. Inner sepals white, narrowly elliptic or lanceolate, 8–14 × 2.5–5.5 mm, apex acuminate. Petals reddish purple, cucullate, much shorter than inner sepals, 3–4 mm, with blunt incurved spur and slight lamina base. Stamens conspicuously prolonged, ca. 8 mm; anthers ca. 2.5 mm; filaments 4.5–5 mm.

#### Distribution and habitat.

*Epimedium dolichostemon* is distributed in Chongqing, northeastern Guizhou, and western Hubei, and usually occurs in forests, thickets, and weedy slopes, at elevations of 800 to 1400 m.

#### Phenology.

*Epimedium dolichostemon* flowers from March to April, and fruits from April to May.

#### IUCN Red List category.

*Epimedium dolichostemon* should be designated as nearly threatened (NT) according to IUCN Red List criteria (IUCN 2013), because of exploitation for medicinal use.

#### Notes.

He and Xu (2003) recognized *Epimedium dewuense* as a new species based only on the comparison with *Epimedium sagittatum* but not with *Epimedium dolichostemon*. According to the protologue of *Epimedium dolichostemon* and *Epimedium dewuense*, the two species can be distinguished by the shape and indumentum of leaflets: *Epimedium dolichostemon* bears narrowly ovate leaves glabrous on both sides, while *Epimedium dewuense* has wider leaves pubescent on both sides (Stearn 1990; He and Xu 2003). Based on investigations in herbaria and the field, morphological variation of leaflets between the two species is continuous, and *Epimedium dewuense* is here treated as a new synonym of *Epimedium dolichostemon*.

#### Specimens examined.

**China. Chongqing:** Shizhu, *Y. Chen 59* (CDBI). **Guizhou:** Dejiang, *B.L. Guo A78* (IMD), *Y.J. Zhang 163* (HIB), *249* (HIB), *339* (HIB); Wuchuan, *S.Z. He* et al. *405001* (GZTM), *405002* (GZTM), *Y.J. Zhang 165* (HIB), *170* (HIB), *171* (HIB). **Hubei:** Jianshi, *Y.M. Wang 15* (HIB); Lichuan, *B.L. Guo A51* (IMD), *89008* (IMD), *89010* (IMD), *H.J. Li 11029* (HIB), *R.H. Huang 3725* (HIB), *Y.J. Zhang 247* (HIB), *248* (HIB), *334* (HIB), *Z.E. Zhao & Y.H. Wang 3201* (HIB); Xianfeng, *X.S. Zou 74009* (HIB).

### Epimedium borealiguizhouense

Taxon classificationPlantaeRanunculalesBerberidaceae

S.Z.He & Y.K.Yang

Epimedium borealiguizhouense S.Z. He & Y.K. Yang, J. Pl. Resourc. Environ. 2(4): 51. 1993, as “baieali-guizhouense”. Type:—China. Guizhou: Yanhe, nearby brooklet in valley, alt. 300-500 m, 11 Apr. 1993, *S.Z. He 93001* (holotype, GZTM!).
Epimedium sagittatum Maxim. var. oblongifoliolatum Z. Cheng, Flora Hubeiensis, 1: 406. 2001. Syn. nov. Type:—China. Hubei: Enshi, alt. 400 m, 24 Apr. 1974, *X.S. Zou 74004* (holotype, HIB!).

#### Description.

Herbs 40–80 cm tall. Rhizome compact. Leaves basal and cauline, trifoliolate; leaflets lanceolate or narrowly lanceolate, 13–18 × 2.5–4 cm, apex long acuminate, margin spinous-serrate, base shallowly cordate, terminal leaflet with subequal rounded lobes, lateral leaflets much oblique, inner lobes small, rounded or almost truncate, outer lobes larger, triangle, acuminate or acute, leathery, adaxially glabrous, abaxially densely strigose, densely pubescent, lanose, or glabrous. Flowering stem with 2 opposite or rarely 3 alternate leaves. Inflorescence paniculate, 30–40 cm long, many (up to 200) flowered, glabrous. Flowers small, 6–10 mm in diam. Outer sepals purplish, small, soon falling. Inner sepals white, ovate, 3–4 × 1.5–2.2 mm, apex acute. Petals yellow, calceiform, 2.2–3.2 mm. Stamens ca. 4 mm; anthers ca. 2.5 mm; filaments ca. 1.5 mm. Capsules ca. 1 cm.

#### Distribution and habitat.

*Epimedium borealiguizhouense* is distributed in south central Chongqing, northeast Guizhou, western Hubei, and western Hunan. It usually occurs in forests, thickets, weedy slopes, and streamsides in valleys, at elevations of 300 to 800 m.

#### Phenology.

*Epimedium borealiguizhouense* flowers from March to April, and fruits from April to May.

#### IUCN Red List category.

*Epimedium borealiguizhouense* should be designated as nearly threatened (NT) according to IUCN Red List criteria (IUCN 2013), because of exploitation for medicinal use.

#### Notes.

*Epimedium borealiguizhouense* is a member of series *Brachycerae* Stearn. Within this series, it is particularly similar to *Epimedium myrianthum* Stearn, but both are obviously different in the shape of the leaflets: *Epimedium borealiguizhouense* bears lanceolate or narrowly lanceolate leaflets with the base of terminal leaflet shallowly cordate, the lateral leaflet is much more oblique with the inner lobe much smaller and rounded or almost truncate and the outer lobe triangular and acuminate or acute, while *Epimedium myrianthum* usually has narrowly ovate leaflets with the base moderately cordate with a narrow sinus and the lateral leaflet is oblique with the lobes rounded or acute.

*Epimedium borealiguizhouense* was previously known only from the type locality, Yanhe, Guizhou. According to herbarium and field investigations, new localities of the species are recorded for Chongqing, Hubei, and Hunan. Furthermore, the indumentum on the abaxial surface of the leaflets varies from densely strigose to densely pubescent, lanose to glabrous. In addition, Epimedium sagittatum var. oblongifoliolatum published in Flora Hubeiensis (Fu 2001) is the same species as *Epimedium borealiguizhouense* and is synonymized here.

#### Specimens examined.

**China. Chongqing:** Changshou, *B.L. Guo 522* (IMD), *Y.J. Zhang 400* (HIB), *420* (HIB); Dianjiang, *Dianjiang Med. Pl. Exped. 82* (SM), *Y.J. Zhang 421* (HIB); Fengdu, *J.A. Wang & Y.X. Wang 49* (CDBI); Hechuan, *Hechuan Med. Pl. Exped. 731* (SM); Pengshui, *Anon. 214* (SM), *F.T. Pu & Y.L. Cao 255* (CDBI), *Y.J. Zhang 419* (HIB). **Guizhou:** Yanhe, *B.L. Guo A80* (IMD), *S.Z. He 93007* (GZTM), *93010* (IMD), *Y.J. Zhang 231* (HIB), *301* (HIB), *325* (HIB). **Hubei:** Enshi, *B.L. Guo & X.Z. Luo 89011* (IMD), *89014* (IMD), *Y.J. Zhang 20* (HIB), *62* (HIB), *399* (HIB), *415* (HIB); Laifeng, *Y.J. Zhang 417* (HIB); Xianfeng, *X.S. Zou 74011* (HIB). **Hunan:** Baojing, *Y.J. Zhang 411* (HIB).

## Supplementary Material

XML Treatment for Epimedium acuminatum

XML Treatment for Epimedium membranaceum

XML Treatment for Epimedium leptorrhizum

XML Treatment for Epimedium dolichostemon

XML Treatment for Epimedium borealiguizhouense
